# Association of sleep quality during pregnancy with stress and depression: a prospective birth cohort study in China

**DOI:** 10.1186/s12884-019-2583-1

**Published:** 2019-11-27

**Authors:** Ming Gao, Jiajin Hu, Liu Yang, Ning Ding, Xiaotong Wei, Lin Li, Lei Liu, Yanan Ma, Deliang Wen

**Affiliations:** 1 Department of Social Medicine, Institute of health sciences, China Medical University, Shenyang, 110122 Liaoning China; 2 Research Center of China Medical University Birth Cohort, China Medical University, Shenyang, Liaoning China; 3 Department of Obstetrics and Gynecology, Shenyang Maternity and Child Health Hospital, Shenyang, Liaoning China; 4 Curriculum and Teaching Research Office, Research Center of Medical Education, China Medical University, Shenyang, Liaoning China; 5 Department of Developmental Pediatrics, Shengjing Hospital of China Medical University, Shenyang, Liaoning China; 6 Department of Epidemiology and Health Statistics, School of Public Health, China Medical University, Shenyang, 110122 Liaoning China

**Keywords:** Sleep quality, Stress, Depression, Pregnancy, China

## Abstract

**Background:**

The sleep quality of pregnant women in the third trimester is related to mental health. However, there is still a lack of large-scale cohort research exploring this relationship in the second trimester. Thus, we assessed the associations of sleep quality during the second trimester with antenatal stress and antenatal and postnatal depression.

**Methods:**

We examined 1152 pregnant women from a prospective cohort study in China to assess the associations of sleep quality in the second trimester with antenatal stress, antenatal depression, and postnatal depression. We used linear regression models and logistic regression models to examine the associations of sleep quality (Pittsburgh Sleep Quality Index [PSQI]) during pregnancy with perinatal stress (Pregnancy Pressure Scale [PPS]) and depression (Edinburgh Postnatal Depression Scale [EPDS]) status. We further assessed the relationship in groups divided according to maternal age.

**Results:**

PSQI scores were positively associated with antenatal PPS scores (β: 1.52, 95% confidence interval [CI]: 1.28, 1.76), antenatal EPDS scores (β: 0.68, 95% CI: 0.58, 0.78), and postpartum EPDS scores (β: 0.51, 95% CI: 0.38, 0.64). Poor sleep quality (PSQI scores ≥5) was associated with antenatal stress status (odds ratio [OR]: 2.60, 95% CI: 1.79, 3.77), antenatal depression status (OR: 3.42, 95% CI: 2.48, 4.72), and postpartum depression status (OR: 2.40, 95% CI: 1.58, 3.64) after adjusting maternal age, BMI, gestational age, smoking, educational level, annual household income and social support. The association of poor sleep quality (PSQI scores ≥5) in the second trimester with postnatal depression status was significant among women more than or equal to 30 years old (OR: 4.12, 95% CI: 2.18, 7.78) but not among women less than 30 years old after adjusting covariates above.

**Conclusion:**

Poor sleep quality in the second trimester among Chinese pregnant women is associated with stress and depression symptoms. Strategies to boost sleep quality should be considered during prenatal health care to improve women’s mental health status.

## Background

The poor mental health of perinatal women is a global challenge [1, 2]. Worldwide, the prevalence of perinatal depression is 11.9%, and women in low- and middle-income countries have a higher prevalence of perinatal depression than women in high-income countries [1]. In China, the prevalences of antenatal stress and perinatal depression are approximately 90 and 10%, respectively [3–5]. Perinatal stress and depression may lead to preterm birth, low birth weight [6], and an emotional and language developmental delay in offspring [7]. Moreover, severe depression is the third leading cause of years lived with disability among Chinese people [8].

Previous work has shown that sleep quality during pregnancy is associated with antenatal stress and perinatal depression [9, 10]. However, the associations between sleep quality in the second trimester and mental health are inconsistent [5, 11–13]. According to the FinnBrain Birth Cohort Study [14], the effect of sleep disturbances on depressive symptoms is only present in the third trimester, and not in the second trimester. However, Okun et al. [15] suggested that sleep disturbances are associated with depressive symptoms in both the second and third trimesters. Additionally, the LIFE Child Study [10] indicated that poor sleep quality can cause stress in pregnant women, whether in the second or third trimester of pregnancy. Furthermore, few studies have examined the age-specific association of sleep quality with the mental health of perinatal women. There is still a lack of large cohort studies in northern China to explore the effects of sleep quality on perinatal depression and antenatal stress.

According to previous work [16], sleep quality worsens with gestational age and the age of the pregnant woman. Thus, we considered that it would be useful to further explore the age-specific associations of sleep quality with mental health after adjustment for gestational age.

Thus, to address these gaps, we investigated the associations of sleep quality during pregnancy with antenatal stress and perinatal depression in a prospective pre-birth cohort study in China. We hypothesized that: (1) poor sleep quality during the second trimester would be associated with psychological stress and depression; and (2) the associations of sleep quality with mental health would differ by the age of the pregnant woman.

## Methods

### Study population and design

The research sample was derived from the Born in Shenyang Cohort Study (BISCS), which follows mothers and children from pregnancy to 12 months postpartum. The aim of the BISCS is to explore and verify risk factors for maternal and infant health. We recruited participants from April 2017 to September 2017. Pregnant women were enrolled from 54 hospitals and community health care centers providing antenatal and maternity care in the urban areas of Shenyang. The eligibility criteria included: (1) singleton pregnancy; (2) second trimester (14–27 weeks) at enrollment; and (3) no plan to move from Shenyang during the subsequent 3 years. All participants provided written information consent, and the study was approved by the ethics committee of China Medical University.

Participants were interviewed in person during the second trimester (mean ± SD: 23.63 ± 3.28 weeks). We also followed up mothers and infants at the child development clinics at an infant age of 3 months. We collected sociodemographic, environmental, behavioral, and clinical information on mothers and children by using standardized questionnaires.

2068 women were invited and 730 refused to participate. In total, 1338 women agreed to participate in the study and 1260 had a live singleton birth. Among the 1260 women with live singleton births, 1152 completed the sleep quality, stress, and depression status questionnaires during the second trimester and 739 underwent postnatal depression assessment 3 months after giving birth. The response rate for the 3-month visit was 65%. In total, 1152 mother-child pairs were included in the present study. Study flow diagram (Additional file 1: Fig. S1) was provided in supplementary file. We compared the characteristics of the 1152 participants included in this study with those excluded. There were no significant differences in demographic indicators between the excluded individuals and those retained.

### Exposure: sleep quality

We assessed sleep quality using the Pittsburgh Sleep Quality Index (PSQI) [17], a validated tool for the measurement of sleep quality in Chinese pregnant women [18, 19]. The PSQI includes a 19-item self-rating questionnaire that assesses sleep quality during the past month. The total score ranges from 0 to 21, with higher scores indicating worse sleep quality. Poor sleep quality was defined as a sum score of ≥5 in accordance with previous studies [19, 20]. The sensitivity and specificity of the questionnaires are 89.6 and 86.5%, respectively.

### Outcome: stress and depression status

We assessed pregnancy stress status using the Pregnancy Pressure Scale (PPS), which is a validated tool for Chinese pregnant women [21]. The PPS includes 30 items valued from 0 to 3 to obtain a score between 0 and 90, with higher scores indicating increased stress status. The cutoff point of a PPS standardized score > 0 reflects a state of stress [21, 22]. We assessed prenatal and postpartum depression status using the Edinburgh Postnatal Depression Scale (EPDS) in accordance with previous studies [23, 24]. The EPDS is a structured 10-item self-report measurement of depression during pregnancy. Items are scored with a value from 0 to 3, which give a sum score of 0 to 30 [23]. It is also validated for screening depression during pregnancy [25]. The cutoff point of an EPDS standardized score ≥ 9 reflects depressive symptomatology in the Chinese population (sensitivity, 80.0%; specificity, 83.0%) [26].

### Covariates

We collected participants’ age, height, weight, educational level, household income, smoking status, gestational age (in weeks), and social support during the second trimester. We treated age (in years) as a continuous variable in multivariate regression analysis. Educational level was classified into four categories (junior high school or lower, senior high school, university, and postgraduate). Annual household income was calculated in Chinese Yuan. Household income was divided into five categories (< ¥10,000, ¥10,000–¥30,000, ¥30,000–¥50,000, ¥50,000–¥70,000, and ≥ ¥70,000). Pre-pregnancy body mass index (BMI) was divided into two categories (< 23 and ≥ 23) in a multilevel logistic model [27]. Smoking status was treated as a dichotomized variable (yes/no). Gestational age at recruitment was treated as a continuous variable. We assessed the social support of pregnant women using the Social Support Rating Scale (SSRS), which has been widely applied to the Chinese population [28]. The SSRS includes 10 items and has a total score of 12 to 65. Higher scores indicate higher levels of social support. The SSRS has high reliability and validity in the Chinese population [29, 30]. The cutoff point of the SSRS is such that scores ≥45 mean high social support; scores below the cutoff mean low social support [31, 32]. Multiple imputation was used to impute missing values for SSRS scores (*n* = 122) and gestational age (*n* = 115). No missing values were observed for any of the other covariates.

### Statistical analysis

We used a t-test and chi-square tests to describe the characteristics of exposures and covariates classified by PSQI scores (< 5 vs. ≥ 5).

Multiple linear regression was used to investigate the associations of PSQI scores with PPS scores and EPDS scores. The following variables were considered potential confounders: maternal age, pre-pregnancy BMI, educational level, household income, pre-pregnancy smoking, gestational age, and social support.

We used logistic regression models to assess the odds ratio (OR) and 95% confidence interval (CI) for stress during pregnancy, antenatal depression, and postnatal depression in relation to sleep quality using PSQI scores of participants < 5 as reference. We conducted crude and adjusted analyses using the following models: Model 1, the crude model; Model 2, adjusted for maternal age, pre-pregnancy BMI, educational level, household income, pre-pregnancy smoking, and gestational age; and Model 3, additionally adjusted for social support based on Model 2. In addition, we conducted stratified analysis classified by women’s age (less than 30 vs. more than or equal to 30 years old).

For sensitivity analyses, multiple imputation was used to impute the missing values (SSRS scores and gestational age), which may be confounders in the study. In addition, we used the age categories of younger than 30, 30 to 35, and older than 35 years old in stratified analyses for comparisons with previous results.

All analyses were conducted with Stata S.E. version 15 (Stata Corp., Texas, TX, USA).

## Results

By the end of 2017, 1152 and 739 participants had completed pregnancy and 3-month postpartum assessments, respectively. Of them, 739 participants (65%) completed all assessments. The missing data analysis showed that there was no significant difference between the excluded samples and those retained as test variables (*p* = 0.326). The participants’ demographic characteristics are shown in Table 1. There were no significant differences in age, gestational age, BMI, smoking, or social support between the two sleep quality groups (PSQI < 5 vs. PSQI ≥5).

**Table 1:** Characteristics of participants in the Born in Shenyang Cohort Study (BISCS)

Variable	PSQI^a^ scores < 5 N (%) or mean ± SD	PSQI scores ≥5 N (%) or mean ± SD	*P*
Age (years)			0.801
< 30	240 (20.83)	317 (27.52)	
≥ 30	252 (21.88)	343 (29.77)	
Gestational age (weeks)	23.48 ± 3.20	23.76 ± 3.33	0.177
BMI (kg/m^2^)			0.632
< 23	325 (28.21)	427 (37.07)	
≥ 23	167 (14.50)	233 (20.23)	
Smoking			0.626
No	465 (40.36)	628 (54.51)	
Yes	27 (2.34)	32 (2.78)	
Educational level			0.033^*^
Junior high school or lower	51 (4.43)	41 (3.56)	
Senior high school	84 (7.29)	97 (8.42)	
University	314 (27.26)	457 (39.67)	
Postgraduate	43 (3.73)	65 (5.64)	
Annual household income (CNY) ^b^			0.018^*^
< 10,000	44 (3.82)	96 (8.33)	
10,000–30,000	71 (6.16)	96 (8.33)	
30,000–50,000	143 (12.41)	156 (13.54)	
50,000–70,000	117 (10.16)	140 (12.15)	
> 70,000	117 (10.16)	172 (14.93)	
Social support scores			0.786
< 45	350 (30.38)	481 (41.75)	
≥ 45	87 (7.55)	112 (9.27)	
Missing value^c^	55 (4.77)	67 (5.82)	
Pregnancy stress scores			< 0.001^*^
= 0	91 (7.90)	53 (4.60)	
> 0	401 (34.81)	607 (52.69)	
Antenatal depression			< 0.001^*^
No	431 (37.41)	450 (39.06)	
Yes	61 (5.30)	210 (18.23)	
Postnatal depression			< 0.001^*^
No	283 (24.57)	317 (27.52)	
Yes	40 (3.47)	99 (8.59)	
Missing value^d^	169 (14.67)	244 (21.18)	

a. PSQI: Pittsburgh Sleep Quality Index

b. CNY: Chinese Yuan (1 Chinese Yuan = 0.14 US Dollar)

c. Missing values were caused by invalid data or incorrect form completion

d. Missing values were caused by loss to follow-up after delivery

^*^ Statistically significant at α = 0.05

**Fig. 1:**
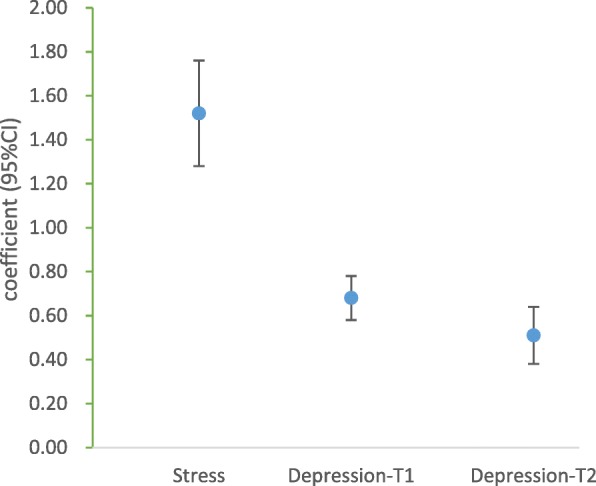
Associations of Pittsburgh Sleep Quality Index (PSQI) scores with Pregnancy Stress Scale (PPS) and Edinburgh Postnatal Depression Scale (EPDS) scores among Born in Shenyang Cohort Study (BISCS) participants (β, 95% confidence interval), adjusted for age, BMI, gestational age, smoking, educational level, annual household income, and social support. Depression-T1: antenatal depression. Depression-T2: postnatal depression

Figure 1 shows the associations of sleep with stress and depression by multiple linear regression. After adjustment for age, BMI, gestational age, smoking, educational level, income level, and social support, higher PSQI scores were associated with higher antenatal PPS scores (β: 1.52, 95% CI: 1.28, 1.76), antenatal EPDS scores (β: 0.68, 95% CI: 0.58, 0.78), and postpartum EPDS scores (β: 0.51, 95% CI: 0.38, 0.64).

The associations of sleep quality with stress and depression status by logistic regression are presented in Table 2. The results of model 1 suggested that participants with poor sleep quality are more likely to suffer from stress during pregnancy (OR: 2.60, 95% CI: 1.81, 3.73), antenatal depression (OR: 3.30, 95% CI: 2.41, 4.51), and postpartum depression (OR: 2.21, 95% CI: 1.48, 3.30) compared with those with good sleep quality. After covariate adjustment, the final model revealed similar results (OR: 2.60, 95% CI: 1.79, 3.77; OR: 3.42, 95% CI: 2.48, 4.72; and OR: 2.40, 95% CI: 1.58, 3.64, respectively).

**Table 2:** Associations of sleep quality with stress and depression status among Born in Shenyang Cohort Study (BISCS) participants

PSQI ^a^ ≥ 5 (Ref: PSQI < 5)	Stress [OR (95%CI)] (*n* = 1152)	Depression-T1 ^b^ [OR (95%CI)] (n = 1152)	Depression-T2 ^c^ [OR (95%CI)] (*n* = 739)
Model 1	2.60 (1.81,3.73)	3.30 (2.41,4.51)	2.21 (1.48,3.30)
Model 2	2.60 (1.80,3.76)	3.43 (2.49,4.72)	2.41 (1.59,3.65)
Model 3	2.60 (1.79,3.77)	3.42 (2.48,4.72)	2.40 (1.58,3.64)

a. PSQI: Pittsburgh Sleep Quality Index

b. Depression-T1: antenatal depression

c. Depression-T2: postnatal depression

Model 1: crude model. Model 2: adjusted for age, BMI, gestational age, smoking, educational level, and annual household income. Model 3: Model 2 + social support

Table 3 presents the comparison of the associations of sleep quality with stress and depression status between the two different age groups (≥ 30 vs. < 30 years old). Older pregnant women were at higher risk of having antenatal stress and antenatal depression symptoms than younger pregnant women in all models. The associations of sleep quality with postnatal depression status were statistically significant only among pregnant women ≥30 years old.

**Table 3:** Associations of sleep quality with stress and depression status among Born in Shenyang Cohort Study (BISCS) participants classified by age

PSQI ^a^ ≥ 5 (Ref: PSQI < 5)	Stress [OR (95% CI)]		Depression-T1 ^b^ [OR (95% CI)]^a^		Depression-T2 ^c^ [OR (95% CI)]^b^	
	<30 years old (*n* = 557)	≥30 years old (*n* = 595)	<30 years old (*n* = 557)	≥30 years old (*n* = 595)	<30 years old (*n* = 345)	≥30 years old (*n* = 394)
Model 1	1.77 (1.08,2.90) ^*^	4.00 (2.31,6.91) ^*^	3.18 (2.06,4.88) ^*^	3.48 (2.19,5.52)^*^	1.34 (0.77,2.34)	3.69 (2.01,6.76) ^*^
Model 2	1.75 (1.05,2.90) ^*^	4.12 (2.34,7.23) ^*^	3.29 (2.12,5.12) ^*^	3.52 (2.20,5.63) ^*^	1.48 (0.82,2.69)	3.93 (2.11,7.35) ^*^
Model 3	1.75 (1.05,2.91) ^*^	4.15 (2.35,7.33) ^*^	3.28 (2.11,5.11) ^*^	3.53 (2.20,5.67) ^*^	1.46 (0.80,2.64)	4.12 (2.18,7.78) ^*^

a. PSQI: Pittsburgh Sleep Quality Index

b. Depression-T1: antenatal depression

c. Depression-T2: postnatal depression

^*^ Statistically significant at α = 0.05

Model 1: crude model. Model 2: adjusted for age, BMI, gestational age, smoking, educational level, and annual household income. Model 3: Model 2 + social support

The associations of sleep quality with stress and depression status without multiple imputation are shown in Additional file 2: Table S1. The OR results in all models were roughly the same. The age categories of participants younger than 30, 30 to 35, and older than 35 years in stratified analyses are shown in Additional file 2: Table S2, whereas the age categories of participants younger than 35 and older than 35 years are shown in Additional file 2: Table S3. Similar conclusions were found using another classification criterion, except that the sample size of the older than 35 years group was insufficient.

## Discussion

In this birth cohort study, we found that women’s EPDS scores during the second trimester were positively associated with antenatal PPS scores and antenatal and postnatal EPDS scores. Poor sleep quality was associated with stress during pregnancy and antenatal and postnatal depression. In the stratified analysis, we found that the association of sleep quality in the second trimester with postnatal depression was specific to the<30-year-old group and was not present in the older group. To our knowledge, this is the first prospective study to assess age-specific associations of sleep quality with stress and depression in China.

Our findings add to the evidence in the literature [10, 14] on the relationship between sleep quality during pregnancy and antenatal or postnatal psychology. Although previous work [33] did examine the association of sleep quality with psychology in Chinese pregnant women, our study is the first to assess both stress and depression in the second trimester and to follow the participants to 3 months postpartum. We found that the association of sleep quality in the second trimester with postnatal depression status varied according to age, which could help in the development of intervention strategies for mental health among pregnant women in China, especially among elderly pregnant women.

Our findings showed that PSQI scores were positively associated with PPS scores after adjustment for potential confounders. Our findings are in concordance with previous work in China [11], which found that PSQI scores were cross-sectionally correlated with PPS scores during pregnancy. Similarly, according to Okun et al. [34], pregnant women who are sleep deficient during the first trimester report more perceived stress and depressive symptoms. Yu et al. [33] reported that the association between sleep and mental status is strongest in the second trimester compared with the first and third trimesters. This may be because pregnant women in the second trimester are free from nausea and vomiting or fear of delivery. Our participants were in the second trimester, which supports this view. The effect of sleep quality on stress status may be associated with arginine vasopressin, which is involved in the stress response and circadian regulation of the sleep-wake cycle [35].

Our findings suggest that higher PSQI scores are associated with higher antenatal EPDS scores. Our findings are in accordance with the results of the Zhoushan Pregnant Women Cohort (ZPWC) [33] and Leipzig Research Center for Civilization Diseases (LIFE) Child BIRTH study (“LIFE Child Study”) [10], which found that sleep quality during pregnancy was associated with antenatal depression in cross-sectional analyses. However, our findings were inconsistent with those of the FinnBrain Birth Cohort Study [14], which only found an association between sleep and depressive symptoms in the third trimester, and not the second. The difference may be explained by the different tools used for measuring sleep quality (they used the Basic Nordic Sleep Questionnaire) or the criteria used to define poor sleep quality.

As for postnatal depression, our findings are in agreement with those of Tham et al. [36], who also reported that PSQI scores during pregnancy are associated with EPDS scores 3 months postpartum. Although they further adjusted for antenatal depressive symptoms in order to evaluate a pure effect, we wanted to assess the complete effect of sleep quality during pregnancy on postpartum depression. Similarly, the CHILD-SLEEP study indicated that sleeping problems of pregnant women in the third trimester were associated with increased depressive symptoms at 3 months postpartum [37]. These findings indicated that the effect of poor sleep quality during pregnancy on psychology would persist until delivery. A possible mechanism could be that sleep quality during pregnancy increases the risk of antenatal depressive symptoms and that antenatal depressive symptoms during pregnancy could predict postpartum depression [38].

However, in stratified analyses, the associations of sleep quality during pregnancy with postnatal depression status were statistically significant only among pregnant women ≥30 years old. Previous research indicated that the sleep quality of pregnant women worsens with age [16]. In addition, a meta-analysis of quantitative sleep parameters indicated that sleep quality naturally decreases with age [39]. We assume that pregnant women ≥30 years old experience worse sleep quality than women < 30 years old throughout the entire pregnancy and after delivery, which increases the risk of postpartum depression. In addition, our results showed that pregnant women ≥30 years old are more likely to suffer stress and depressive symptoms during pregnancy, which also probably increased the risk of postpartum depression among pregnant women ≥30 years old.

The strength of our study includes the prospective design based on a community population and a relatively large sample size. In addition, our research recruited participants in the second trimester of pregnancy, a phase that receives little attention and has fewer confounders of the association of sleep quality with mental health [33].

Our work also has several limitations. First, sleep quality was based on self-reports and lacked an objective measurement of sleep quality. Importantly, previous studies indicated that antenatal and postnatal emotional distress is associated with subjective assessments of sleep quality and not an objective measurement (wrist actigraphy) [40, 41]. Second, we did not measure sleep quality in the third trimester and after delivery and thus failed to compare the association of sleep quality at different phases on mental health. Third, we did not measure postnatal stress. Fourth, there may be residual confounders that we did not take into account. Lastly, our findings are based on a regional population and may not be generalizable to other settings.

## Conclusions

In summary, sleep quality during pregnancy is associated with antenatal stress and antenatal and postnatal depression. Although the effect of sleep quality on postnatal depression may only exist in elderly pregnant women (≥ 30 years), the impact of sleep on mental stress and depression status during pregnancy should not be ignored. More attention needs to be paid to the mental health of pregnant women and sleep interventions may be a good way to solve the problem.

## Supplementary information

**Additional file 1: Figure S1.** Study flow diagram. a. PSQI: Pittsburgh Sleep Quality Index. b. PPS: Pregnancy Pressure Scale. c. EPDS: Edinburgh Postnatal Depression Scale.

**Additional file 2: Table S1.** Association of sleep quality with stress and depression status among “Born in Shenyang Cohort Study” (BISCS) participants (without multiple imputation). **Table S2.** Association of sleep quality with stress and depression status classified by age (< 30, 30–35, ≥ 35) among “Born in Shenyang Cohort Study” (BISCS) participants. **Table S3.** Associations of sleep quality with stress and depression status among Born in Shenyang Cohort Study (BISCS) participants classified by age (<35, ≥ 35).

## Data Availability

The datasets used for analysis during the current study are available from the corresponding author upon request.

## Abbreviations

BISCS: Born in Shenyang Cohort Study
EPDS: Edinburgh Postnatal Depression Scale
PPS: Pregnancy Pressure Scale
PSQI: Pittsburgh Sleep Quality Index
SSRS: Social Support Rating Scale
