# Supplementary material for: Association of sleep quality during pregnancy with stress and depression: a prospective birth cohort study in China
Source: BMC Pregnancy Childbirth. 2019 Nov 27;19:444. doi: 10.1186/s12884-019-2583-1 (PMC6882237; doi:10.1186/s12884-019-2583-1)
Supplement: Supplementary file 1 — Additional file 1: Figure S1. Study flow diagram. a. PSQI: Pittsburgh Sleep Quality Index. b. PPS: Pregnancy Pressure Scale. c. EPDS: Edinburgh Postnatal Depression Scale. [file 12884_2019_2583_MOESM1_ESM.docx]

2,068 women invited to participate at 21-24 weeks gestation

N=730 refused to participate

1,338 women agreed to participate

N=36 stillbirths or miscarriages

N=42 withdrew

1,260 women with single live birth

N=41, missing data on PSQI ^a^

N=49, missing data on PPS ^b^

N=18, missing data on EPDS ^c^ at the second trimester

1152 eligible women at the second trimester

739 eligible women 3 months postpartum

N=413, missing data on EPDS 3 months postpartum
