# Supplementary material for: Association of sleep quality during pregnancy with stress and depression: a prospective birth cohort study in China
Source: BMC Pregnancy Childbirth. 2019 Nov 27;19:444. doi: 10.1186/s12884-019-2583-1 (PMC6882237; doi:10.1186/s12884-019-2583-1)
Supplement: Supplementary file 2 — Additional file 2: Table S1. Association of sleep quality with stress and depression status among “Born in Shenyang Cohort Study” (BISCS) participants (without multiple imputation). Table S2. Association of sleep quality with stress and depression status classified by age (< 30, 30–35, ≥ 35) among “Born in Shenyang Cohort Study” (BISCS) participants. Table S3. Associations of sleep quality with stress and depression status among Born in Shenyang Cohort Study (BISCS) participants classified by age (<35, ≥ 35). [file 12884_2019_2583_MOESM2_ESM.docx]

**Table S1.** Association of sleep quality with stress and depression status among “Born in Shenyang Cohort Study” (BISCS) participants (without multiple imputation)

| PSQI ^a^ ≥5  (Ref: PSQI<5) | Stress (OR (95%CI))  (n=934) | Depression-T1^b^ (OR (95%CI))  (n=934) | Depression-T2 ^c^ (OR (95%CI))  (n=601) |
| --- | --- | --- | --- |
| Model1 | 2.69(1.79,4.02) | 3.14(2.23,4.43) | 2.14(1.38,3.33) |
| Model2 | 2.74(1.81,4.14) | 3.20(2.26,4.54) | 2.32(1.47,3.66) |
| Model3 | 2.76(1.82,4.19) | 3.21(2.26,4.57) | 2.29(1.45,3.62) |

a. PSQI : Pittsburgh Sleep Quality Index. b. Depression-T1: antenatal depression. c. Depression-T2: postnatal depression.

Model 1: crude model. Model 2: adjusted for age, BMI, gestational weeks, smoking, educational level and annual household income. Model 3: Model 2 + social support.

**Table S2.** Association of sleep quality with stress and depression status classified by age (＜ 30, 30 - 35, ≥ 35 ) among “Born in Shenyang Cohort Study” (BISCS) participants

| PSQI ^a^ ≥5  (Ref: PSQI<5) | | Stress (OR (95%CI)) | | | Depression-T1 ^b^ (OR (95%CI)) | | | | Depression-T2 ^c^ (OR (95%CI)) | | | | |
| --- | --- | --- | --- | --- | --- | --- | --- | --- | --- | --- | --- | --- | --- |
|  |  | ＜30 years old  (n=557) | 30-35 years old  (n=420) | ≥35 years old  (n=175) | | ＜30 years old  (n=557) | 30-35 years old  (n=420) | ≥35 years old  (n=175) | | ＜30 years old  (n=345) | 30-35 years old  (n=280) | ≥35 years old  (n=114) |  |
| Model1 | 1 | 1.77(1.08,2.90)^*^ | 4.11(2.10,8.05)^*^ | 3.81(1.48,9.82)^*^ | | 3.18(2.06,4.88)^*^ | 4.03(2.33,6.96)^*^ | 2.37(0.99,5.64) | | 1.34(0.77,2.34) | 5.75(2.60,12.73)^*^ | 1.51(0.56,4.09) |  |
| Model2 | 1 | 1.75(1.05,2.90)^*^ | 4.27(2.14,8.52)^*^ | 3.99(1.43,11.15)^*^ | | 3.29(2.12,5.12)^*^ | 4.18(2.39,7.30)^*^ | 2.24(0.89,5.60) | | 1.48(0.82,2.69) | 5.95(2.61,13.57)^*^ | 1.42(0.49,4.15) |  |
| Model3 | 1 | 1.75(1.05,2.91)^*^ | 4.13(2.06,8.29)^*^ | 4.60(1.58,13.39)^*^ | | 3.28(2.11,5.11)^*^ | 4.07(2.32,7.13)^*^ | 2.39(0.94,6.09) | | 1.46(0.80,2.64) | 6.04(2.62,13.93)^*^ | 1.66(0.54,5.15) |  |

a. PSQI : Pittsburgh Sleep Quality Index. b. Depression-T1: antenatal depression. c. Depression-T2: postnatal depression. ^*^ statistically significant at α=0.05 level.

Model 1: crude model. Model 2: adjusted for age, BMI, gestational weeks, smoking, educational level and annual household income. Model 3: Model 2 + social support.

**Table S3.** Associations of sleep quality with stress and depression status among Born in Shenyang Cohort Study (BISCS) participants classified by age (＜35, ≥ 35)

| PSQI ^a^ ≥ 5  (Ref: PSQI < 5) | Stress [OR (95%CI)] | | Depression-T1 ^b^ [OR (95%CI)]^a^ | | Depression-T2 ^c^ [OR (95%CI)]^b^ | |
| --- | --- | --- | --- | --- | --- | --- |
|  | ＜35 years old  (n=977) | ≥35 years old  (n=175) | ＜35 years old  (n=977) | ≥35 years old  (n=175) | ＜35 years old  (n=625) | ≥35 years old  (n=114) |
| Model 1 | 2.43(1.64,3.59) ^*^ | 3.81(1.48,9.82) ^*^ | 3.48(2.48,4.88) ^*^ | 2.37(0.99,5.64) | 2.37(1.53,3.67) ^*^ | 1.51(0.56,4.09) |
| Model 2 | 2.43(1.63,3.61) ^*^ | 3.99(1.43,11.15)^*^ | 3.58(2.54,5.05) ^*^ | 2.24(0.89,5.60) | 2.58(1.64,4.07) ^*^ | 1.42(0.49,4.15) |
| Model 3 | 2.39(1.60,3.57) ^*^ | 4.60(1.58,13.39) ^*^ | 3.55(2.52,5.00) ^*^ | 2.39(0.94,6.09) | 2.54(1.61,4.01) ^*^ | 1.66(0.54,5.15) |

a. PSQI: Pittsburgh Sleep Quality Index.

b. Depression-T1: antenatal depression.

c. Depression-T2: postnatal depression.

^*^ Statistically significant at α=0.05.

Model 1: crude model. Model 2: adjusted for age, BMI, gestational weeks, smoking, educational level and annual household income. Model 3: Model 2 + social support.
